# TSIDER1, a short and non-autonomous Salivarian trypanosome-specific retroposon related to the ingi6 subclade

**DOI:** 10.1016/j.molbiopara.2011.05.007

**Published:** 2011-09

**Authors:** Frédéric Bringaud, Matthew Berriman, Christiane Hertz-Fowler

**Affiliations:** a Centre de Résonance Magnétique des Systèmes Biologiques (RMSB), UMR 5536, Université Bordeaux Segalen, CNRS, 146 rue Léo Saignat, 33076 Bordeaux, France; b Wellcome Trust Sanger Institute, Wellcome Trust Genome Campus, Hinxton, Cambridgeshire CB10 1SA, UK

**Keywords:** SIDER, Short Interspersed DEgenerate Retroposons, DIRE, Degenerate Ingi/L1Tc-Related Element, African trypanosomes, Ingi, Retroposon, Non-LTR retrotransposon, Non-autonomous, SIDER

## Abstract

Retroposons of the ingi clade are the most abundant transposable elements identified in the trypanosomatid genomes. Some are long autonomous elements (ingi, L1Tc) while others, such as RIME and NARTc, are short non-coding elements that parasitize the retrotransposition machinery of the active autonomous ones for their own mobilization. Here, we identified a new family of short non-autonomous retroposons of the ingi clade, called TSIDER1, which are present in the genome of Salivarian (African) trypanosomes, *Trypanosoma brucei*, *T. congolense* and *T. vivax*, but absent in the *T. cruzi* and *Leishmania* spp. genomes and, as such, TSIDER1 is the only retroposon subfamily conserved at the nucleotide level between African trypanosome species. We identified three TvSIDER1 families within the genome of *T. vivax* and the high level of sequence conservation within the TvSIDER1a and TvSIDER1b groups suggests that they are still active. We propose that TvSIDER1a/b elements are using the Tvingi retrotransposition machinery, as they are preceded by the same conserved pattern characteristic of the ingi6 subclade, which corresponds to the retroposon-encoded endonuclease binding site. In contrast, TcoSIDER1, TbSIDER1 and TvSIDER1c are too divergent to be considered as active retroposons. The relatively low number of SIDER elements identified in the *T. congolense* (70 copies), *T. vivax* (32 copies) and *T. brucei* (22 copies) genomes confirms that trypanosomes have not expanded short transposable elements, which is in contrast to *Leishmania* spp. (∼2000 copies), where SIDER play a role in the regulation of gene expression.

## Graphical abstract

**Graphical abstract:**

Position of African trypanosome-specific TSIDER1 retroposons in the ingi and trypanosomatid phylogenetic trees.

**Figure:**
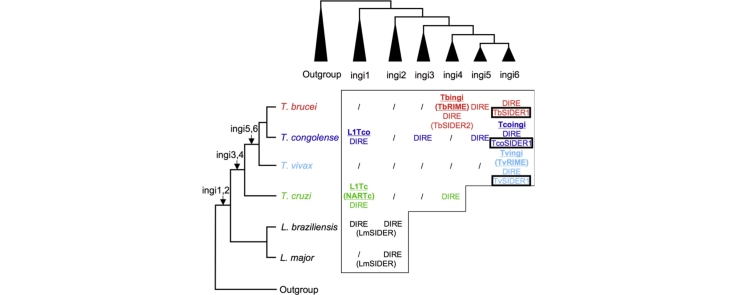

**Highlights:**

► A new retroposon family of the ingi clade, named TSIDER1, was identified. ► TSIDER1 are short degenerate retroposons. ► TSIDER1 is only present in the nuclear genome of African trypanosomes. ► In contrast to *Leishmania*, African trypanosomes have not expanded and domesticated SIDER.

## 1 Introduction

The trypanosomatid family includes some of the most important protist parasites of humans in the genera *Leishmania* and *Trypanosoma*, as well as other species parasitic in a wide variety of vertebrates, invertebrates, ciliates, and plants [1]. Two well-defined major groups have been identified within the genus *Trypanosoma*: (i) the section Stercoraria (also called South-American trypanosomes), including the causative agent of Chagas’ disease (*T. cruzi*), and (ii) the section Salivaria (also called African trypanosomes), including the causative agents of African sleeping sickness (*T. brucei gambiense and T. b. rhodesiense*), along with other African parasites of mammals, such as *T. b. brucei* (*T. brucei*), *T. vivax* and *T. congolense*. The genome of six trypanosomatids has been sequenced and published to date, *i.e*. *T. brucei* [2], *T. b. gambiense* [3], *T. cruzi* [4], *L. major* [5], *L. infantum* [6] and *L. braziliansis* [6]. In addition, a number of ongoing trypanosomatid genome projects are available on line (http://tritrypdb.org/tritrypdb/showXmlDataContent.do?name=XmlQuestions.DataSources), such as two other African trypanosomes *T. congolense* and *T. vivax*. All these aforementioned genomes contain active and/or traces of inactive transposable elements (TE) (for reviews see: [7,8]).

Among the three main classes of eukaryotic transposable elements (TE: retroelements, DNA transposons and Miniature Inverted-repeat Transposable Elements – MITE), only retroelements have been described so far in the trypanosomatid genomes (2–5% of the nuclear genome). Retroelements transpose *via* reverse transcription of an RNA intermediate and are further divided into LTR retrotransposons with long terminal repeats (LTR) and the non-LTR retrotransposons, also called retroposons. Trypanosomatids contain LTR retrotransposons (VIPER), site-specific retroposons (SLACS/CZAR) and retroposons of the ingi clade (ingi/L1Tc) showing a relative site specificity for insertion [7,8]. Retroposons of the ingi clade, which will be further considered herein, contain two categories of active elements (Fig. 1 and Table S1): (i) the long (4736–5419 bp) and autonomous elements originally characterized in *T. brucei* (Tbingi) [9,10] and *T. cruzi* (L1Tc) [11], and subsequently described in *T. vivax* (Tvingi) [12] and *T. congolense* (Tcoingi and L1Tco) [12] and (ii) the short (260–1030 bp) and non-autonomous elements identified in *T. brucei* (TbRIME) [13], *T. cruzi* (NARTc) [14] and *T. vivax* (TvRIME) [12]. The short elements (TbRIME, TvRIME and NARTc) are truncated versions, which are mobilized by the retrotransposition machinery of the corresponding long elements (Tbingi, Tvingi and L1Tc, respectively) [12,15,16]. Consequently, the Tbingi/TbRIME, Tvingi/TvRIME and L1Tc/NARTc associations are considered as pairs of retroposons akin to the human LINE1/Alu, the eel UnaL2/UnaSINE1 and the plant LINE/S1 pairs [17–20].

In addition, the trypanosomatid genomes contain two kinds of degenerate vestigial ingi-related retroposons (Fig. 1 and Table S1): (i) the long elements (∼5000 bp) detected in all the trypanosomatid genomes (DIRE, *D*egenerate *I*ngi/L1Tc-*R*elated *E*lement) [21] and (ii) the small elements (∼600 bp), called SIDER (*S*hort *I*nterspersed *DE*generate *R*etroposon), in *T. brucei* (TbSIDER) and all the *Leishmania* spp. analyzed so far (LmSIDER, LbSIDER, etc.) [22,23]. Interestingly, SIDER retroposons were extensively expanded in the *Leishmania* genomes (∼2000 copies per haploid genomes), where they have been co-opted into a role regulating gene expression [22,24–27]. In contrast, expansion of the SIDER elements has not occurred in the *T. brucei* genome, in which only ∼20 copies have been detected [22]. Here we analyzed SIDER elements in the *T. congolense* and *T. vivax* genomes and report a Salivarian-specific group (TSIDER1) belonging to the ingi6 subclade.

## 2 Materials and methods

### 2.1 Detection of SIDER sequences

BLASTN was used to detect short ingi-related sequences in the *T. congolense* (strain IL3000, version 1 genome release) and *T. vivax* (strain Y486, version 2 genome release) datasets. The *T. congolense* genome assembly consisted of 3181 contigs, totaling 41.8 Mb, while the *T. vivax* assembly contained 10,250 contigs, totaling 47.4 Mb, including 8279 *T. vivax* contigs not assigned to chromosomes [12]. To identify all SIDER sequences, genome contigs were first probed with the 5′-terminal “76–79 bp signature” of Tbingi (79 bp), Tvingi (76 bp), L1Tc (78 bp), LmSIDER2a (79 bp) and LmSIDER2b (77 bp), and iteratively repeated with members of each of the SIDER families that were identified.

### 2.2 Bioinformatics analyses

Alignments were done using the multiple alignment software CLUSTAL X [28], followed by minor manual adjustments using MacClade version 4.06 (Sinauer Associates, Inc). Using online tools available from the European Bioinformatics Institute (EBI, http://srs.ebi.ac.uk/) consensus sequences were generated (CosN) and the percentage of divergence from the consensus sequences calculated (InfoalignN).

## 3 Results

### 3.1 Identification of conserved short non-autonomous retroposons in the African trypanosome genomes

To identify SIDER sequences in the *T. vivax* and *T. congolense* genomes, BLASTN searches were performed with the conserved signature motif present at the 5′-extremity of all trypanosomatid ingi-related elements characterized to date [21]. This approach was previously used to identify 10 TbSIDER1 and 12 TbSIDER2 in the *T. brucei* genome [22], as well as 1858 LmSIDER elements in *L. major* [22] and 1986 in *L. braziliensis* [23]. Here, 70 closely related sequences were identified in the *T. congolense* genome, called TcoSIDER1. Interestingly, the TcoSIDER1 and TbSIDER1 consensus sequences (570 and 571 bp, respectively) show 78% identity, and can therefore be considered as the same family (Fig. 2). As previously observed for TbSIDER1, the TcoSIDER1 sequences are more heterogeneous than the active non-autonomous TbRIME, TvRIME and NARTc retroposons (Fig. 3), suggesting that SIDER1 is not anymore active in the *T. brucei* and *T. congolense* genomes [29]. Indeed, the TcoSIDER1 sequences display 10–30% divergence from their consensus sequences, with a median value of 16% (*cf*. 16% for TbSIDER1), while the potentially active TbRIME, NARTc and TvRIME retroposons have median divergence values of 4%, 2% and 1%, respectively (Fig. 3).

BLASTN searches performed with the TbSIDER1 and TcoSIDER1 consensus sequences, as well as the “76–79 bp signature” motifs, as queries revealed 32 sequences in the *T. vivax* genome. The 576 bp TvSIDER1 consensus sequence shares 75% sequence identity with TbSIDER1 and 85% with TcoSIDER1, respectively (data not shown). The TvSIDER1 sequences could be further subdivided into 3 groups of sequences (Figs. 2 and 3): TvSIDER1a, 7 elements with up to 2% divergence; TvSIDER1b, 4 elements with up to 5% divergence; and TvSIDER1c, a heterogeneous group of 21 elements, which showed 6–40% divergence from their consensus.

The TbSIDER1, TcoSIDER1 and TvSIDER1 elements contain the “76–79 bp signature” and a poly(dA) stretch at their 3′-extremity, a hallmark of retroelements (Figs. 2 and 4). Apart from the “76–79 bp signature”, these SIDER elements (called TSIDER1 for Trypanosome SIDER1) show no sequence similarity with any known trypanosomatid TE, indicating that TSIDER1 is a new family of trypanosomatid retroposons. Furthermore, extensive BLASTN searches against the *T. cruzi* and *Leishmania* spp. genomes, did not identify high-scoring matches, suggesting that TSIDER1 is restricted to the African trypanosomes.

### 3.2 TSIDER1 is still active in the *T. vivax* genome

During retrotransposition, the retroposon-encoded endonuclease performs two asymmetrical single-strand cleavages, leading to a duplication of the residues between both cleavages [30]. The duplicated motif flanking the newly inserted retroposons is called “target site duplication” (TSD). One particularity of the retroposons of the ingi clade is the size conservation of the TSD, which is 12 bp long in the case of Tbingi/TbRIME [15], Tvingi/TvRIME [12], Tcoing/L1Tco [12] and L1Tc/NARTc [16]. A conserved 12 bp motif (>65% identity), which resembles a vestigial TSD was found flanking a proportion of the TSIDER elements: 12/32 TvSIDER1, 20/70 TcoSIDER1 and 1/10 TbSIDER1 (Fig. 5A–C). Interestingly, all members of the TvSIDER1a and TvSIDER1b subfamilies are flanked by identical or near identical 12 bp residues, suggesting that these TvSIDER1 sequences are representatives of still active short retroposon species. This observation is consistent with the high level of nucleotide conservation between members of the TvSIDER1a and TvSIDER1b subfamilies (see above).

Since the non-autonomous TSIDER1 retroposons have no coding capacity, they have to use the retrotransposition machinery encoded by autonomous retroposons for their own retrotransposition. Autonomous/non-autonomous retroposon pairs are well known in many eukaryotes, including the *T. brucei* Tbingi/TbRIME [14–16], *T. vivax* Tvingi/TvRIME [12] and *T. cruzi* L1Tc/NARTc pairs [14–16]. These pairs are often characterized by tandem arrangements of autonomous and non-autonomous elements only separated by a TSD. The *T. vivax* genome contains two TvSIDER1a and one TvSIDER1b sequences tandemly arranged with Tvingi and separated by a 12 bp TSD, suggesting that TvSIDER1 and Tvingi form such a retroposon pairing (Fig. 6).

Three TcoSIDER1 elements are tandemly arranged with Tcoingi or L1Tc, suggesting that TSIDER1 are also active in the *T. congolense* genome (Fig. 6). However, two lines of evidence strongly support the TcoSIDER1 inactivity: firstly, none of the 15 TSD flanking TcoSIDER1 are identical (Fig. 5B); and secondly, as mentioned above, the TcoSIDER1 sequences are highly heterogeneous (Fig. 3). Thus, the numerous point mutations accumulated in the TcoSIDER1 elements and their TSD strongly supports the view that these retroposons have not been mobilized in recent evolutionary history and consequently can no longer be considered active. Similarly, the TbSIDER1 can also being considered as inactive retroposons (Figs. 3 and 5C).

### 3.3 TSIDER1 is related to the ingi6 subclade

Retroposons of the ingi clade show a relative site-specificity for insertion and are preceded by a conserved motif recognized by the element-encoded endonuclease domain, before performing the single-strand nicks at the target site of insertion [12,15,16]. So far, three different conserved sequences have been identified upstream of ingi-related retroposons that are specific for ingi1 (Tcoingi/Tvingi/TvRIME) [12], ingi4 (Tbingi/TbRIME) [15] and ingi6 (L1Tc/NARTc/L1Tco) [12,16] subclades (see Fig. 7). The TvSIDER1, TcoSIDER1 and TbSIDER1 elements are preceded by the ingi6 signature (5′-**TTTT**xxxxx**A**_↑_**A**xxxxxxxxxxx-3′, the arrow indicating the first-strand cleavage site) (Fig. 5A–C), while the ingi1 and ingi4 signatures (see Fig. 7) are not present upstream of the TSIDER1 element. This suggests that the non-autonomous TvSIDER1 and TcoSIDER1 use the machinery of autonomous retroposons of the ingi6 subclades, such as the potentially active Tvingi and Tcoingi, respectively (Fig. 7). The *T. brucei* genome does not contain potentially active autonomous retroposons belonging to the ingi6 subclade, however, inactive and extinct elements of this subclade (TbDIRE) have been identified (see Fig. 7) [12]. Interestingly, these TbDIRE elements are preceded by the ingi6 signature (Fig. S1A), suggesting that TbSIDER1 have used the retrotransposition machinery of this TbDIRE group, which is no longer active.

The *T. brucei* genome contains another family of degenerate short retroposons of the ingi clade (TbSIDER2) [22]. Analysis of the flanking region reveals that TbSIDER2 elements are preceded by the ingi4 signature (5′-**A**xxxxxxx**T**xxx**GT**x**GG**x**T**xxx_↑_x**T**x**T**xx**T**xxxxx-3′) (Fig. 5D), suggesting that TbSIDER2 used the retroposition machinery of long retroposons of the ingi4 subclade, such as Tbingi or TbDIRE belonging to this subclade, before becoming inactive.

## 4 Discussion

By using the “76–79 bp signature” present at the 5′-extremity of all retroposons belonging to the ingi-clade (autonomous or non-autonomous families) as bait, a new family of short retroelements was identified. This ∼570 bp long retroposon family named TSIDER1 (Trypanosome SIDER1) is only present in the genome of the African trypanosomes *T. brucei* (TbSIDER1), *T. congolense* (TcoSIDER1) and *T. vivax* (TvSIDER1). Apart from the conserved “76–79 bp signature”, TSIDER1 shows no similarity at the nucleotide level with other ingi-related retroposons (potentially active elements as well as DIRE). Potentially active short non-autonomous ingi-related retroposons (TbRIME, TvRIME and NARTc) are derived by internal deletion from long autonomous ingi [9,12,14]. This model probably stands for TSIDER1, although its ancestral long ingi-related retroposon is no longer detectable in the African trypanosome genomes. Alternatively, we cannot rule out the possibility that in the African trypanosome ancestor, the “76–79 bp signature” was inserted upstream of the pre-TSIDER sequence to generate a new transposable element (TSIDER1), which was successfully mobilized by the retrotransposition machinery of an ingi-related family.

A comparative analysis of TSIDER1 flanking regions revealed a conserved motif upstream of TbSIDER1, TcoSIDER1 and TvSIDER1, which resembles the putative endonuclease-binding domain preceding Tvingi and Tcoingi of the ingi6 subclade [12]. We concluded that TSIDER1 utilized, or still utilizes, the retrotransposition machinery of active retroposons of the ingi6 subclade, such as Tvingi for TvSIDER1 and Tcoingi in the case of TcoSIDER1. The only long ingi6-related retroposons identified in the *T. brucei* genome are eight TbDIRE elements (called TbDIRE2 in [21]), all preceded by the ingi6 signature (Fig. S1A). Consequently, TbDIRE/TbSIDER1 form an extinct autonomous/non-autonomous retroposon pair, as previously reported for the human LINE2/MIR pair [31]. Moreover, TvDIRE and TcoDIRE are phylogenetically related to the ingi6 subclade and most are preceded by the same **TTTT**xxxxx**A**_↑_**A** motif (Fig. S1B–C). Consequently, one cannot rule out that TcoSIDER1 and TvSIDER1c, which are probably no longer active, used the retrotransposition machinery of these inactive retroposons before their extinction.

Two lines of evidence indicate that two groups of the TSIDER1 family (TvSIDER1a and TvSIDER1b) are still active in the *T. vivax* genome. Firstly, members within each group show a high level of conservation (95–100% identity), indicating that they have been duplicated by retrotransposition quite recently, with very few point mutations. Secondly, these relatively recent events of mobilization are consistent with the presence of conserved 12-bp TSD flanking all the identified TvSIDER1a and TvSIDER1b elements (Fig. 5A). One may consider that the only potentially active autonomous retroposon of the ingi clade detected in the *T. vivax* genome (Tvingi) is responsible for the recent mobilization of TvSIDER1a and TvSIDER1b. This hypothesis is strengthened by the presence of several tandemly arranged TvSIDER1a/TvSIDER1b and Tvingi sequences only separated by the 12-bp TSD (Fig. 6). Tandem arrangement of retroposons has frequently been observed in the *T. brucei* and *T. cruzi* genomes [15,16], as well as in other eukaryotic genomes [20]. Experimental evidence indicates that such tandem arrangements of Human retroposons are the consequence of independent insertion events of different retroelement molecules at the same relative position, due to the recognition of the same target sequence by the retrotransposition machinery [20].

The only conserved sequence between TvSIDER1a/b and Tvingi is the “76–79 bp signature”, suggesting that this N-terminal domain conserved in all retroposons of the ingi clade is essential for mobilization of ingi-related retroelements. Heras and colleagues identified a RNA polII-dependent promoter in the first 77 bp of L1Tc, which is certainly critical for expression and consequently mobilization of ingi-related retroposons [32]. In addition, the “76–79 bp signature” might play a post-transcriptional role. Indeed, none of the Tbingi and L1Tc transcripts contain the spliced leader cap added at the 5′ extremity of all trypanosomatid mRNAs by *trans*-splicing [11,33,34], suggesting that the “76–79 bp signature” might be recognized by ribosomes to initiate translation of the retroposon transcripts. It has also been proposed that the predicted stable RNA structure involving the N-terminal 37 residues of Tbingi and L1Tc might protect the retroposon transcripts against exonucleases [21]. Finally the absence of sequence similarity between TvSIDER1a/b and Tvingi except for the “76–79 bp signature”, but utilization of the Tvingi retrotransposition machinery by TvSIDER1a/b, suggests that the N-terminal signature may play a role in retroelement recognition by the Tvingi endonuclease/reverse transcriptase-containing complex. To test this hypothesis, a retrotransposition assay needs to be developed in trypanosomes, as previously reported for the yeast Ty and human L1 retroposons [35,36].

In the course of this analysis, we identified up to 70 SIDER elements per haploid African trypanosome genomes, while the same analysis conducted on *Leishmania* spp. genomes revealed ∼2000 SIDER copies [22]. This confirms that SIDER expansion and exaptation did not occur in trypanosomes and seems to be a *Leishmania* innovation. A key question is: when did it occur in the Trypanosomatid lineage? Sequencing projects currently underway for trypanosomatid species closely related to *Leishmania* spp., such as *Endotrypanum*, *Crithidia* and *Phytomonas* sp., will certainly help to address this important evolutionary question.

## Figures and Tables

**Figure:**
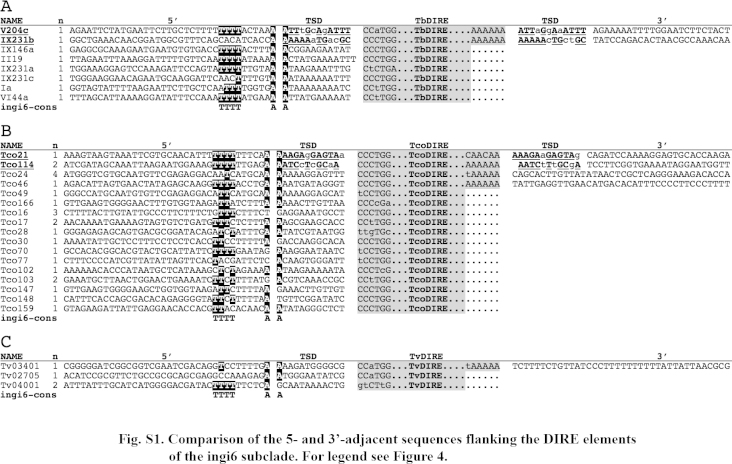


**Fig. 1:**
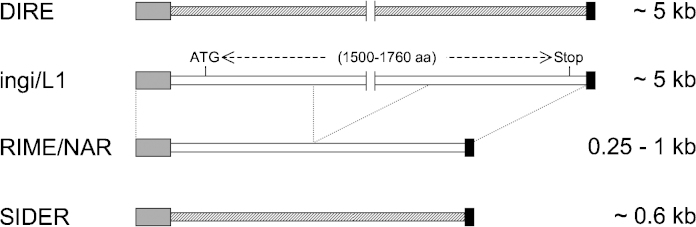
Schematic representation of ingi-related retroposons identified in the trypanosomatid genomes. Potential active retroposons (ingi/L1 and RIME/NAR) and degenerate retroposons (DIRE and SIDER) are represented by white and hatched/grey bars, respectively. The conserved “76–79 bp signatures” and the poly(dA) tails are showed by dark grey and black boxes, respectively. The approximate size of these mobile elements is indicated in the right margin. The long and autonomous ingi/L1 elements are the only retroposons coding for a protein (from 1500 to 1760 aa long) responsible for retrotransposition of themselves or other ingi-related retroposons.

**Fig. 2:**
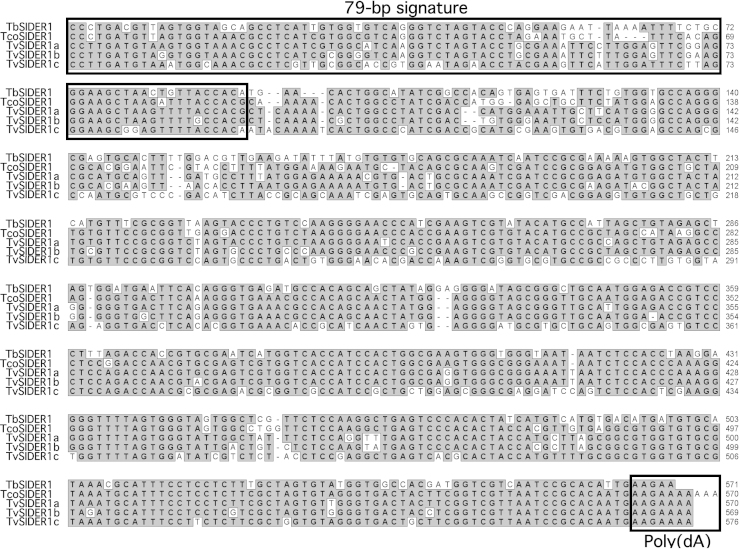
Comparison of TSIDER1 consensus sequences identified in the African trypanosome genomes. The consensus sequences were generated by comparing 10 TbSIDER1 (*T. brucei*), 70 TcoSIDER1 (*T. congolense*), 7 TvSIDER1a, 4 TvSIDER1b and 21 TvSIDER1 (*T. vivax*) copies. Gaps (-) were introduced to maximize the alignments and the residues conserved among at least three sequences are shaded in grey. The “76–79 bp signature” conserved between ingi-related retroposons and the poly(dA) terminal stretch, which is a hallmark of retrotransposons, are boxed.

**Fig. 3:**
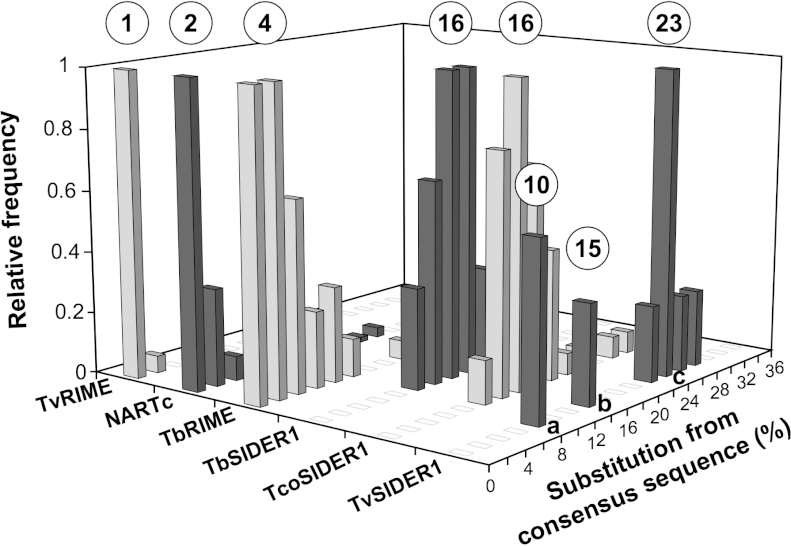
Divergence between members of short non-autonomous retroposons. Bases covered by the *T. cruzi* (NARTc), *T. brucei* (TbRIME and TbSIDER1), *T. congolense* (TcoSIDER1) and *T. vivax* (TvRIME and TvSIDER1) retroposons were sorted by their divergence from their consensus sequence. The consensus sequences determined from the alignment of all retroposons of the same family (TvRIME: 26 copies, NARTc: 115 copies, TbRIME: 70 copies, TbSIDER1: 10 copies, TcoSIDER1: 70 copies, TvSIDER1: 32 copies) approximate the element's original sequence at the time of insertion [29]. The number of retroposons per fraction of 2% divergence is expressed as a fraction of the highest value, for which an arbitrary value of 1 was assigned. The percentage of divergence was calculated using the matching region of the consensus sequences. The circled numbers on the top indicate the median value of each graph. For TvSIDER1 sequences, the median value is indicated for each identified group, labeled a, b and c.

**Fig. 4:**
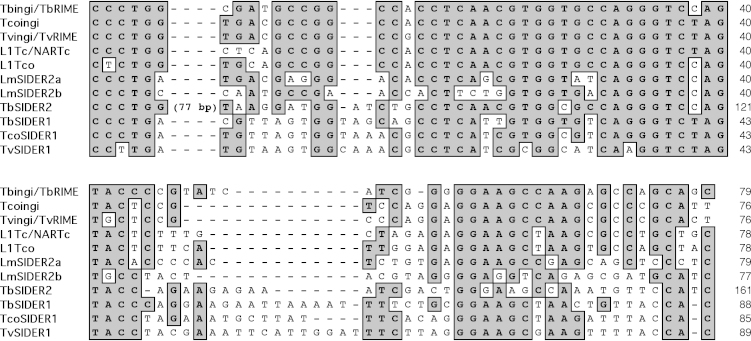
Comparison of the N-terminal “76–79 bp signature” consensus sequences between different trypanosomatid retroposons. This figure shows the alignment of the first 76–161 bp of the consensus sequence generated from alignment of Tbingi/TbRIME (63/70 copies), Tcoingi (27 copies), Tvingi/TvRIME (42/26 copies), L1Tc/NARTc (48/115 copies), L1Tco (9 copies), TbSIDER1 (10 copies), TcoSIDER1 (70 copies) and TvSIDER1 (32 copies) and the two tandemly-repeated “76–79 bp signature” located at the 5′-extremity of LmSIDER2 (LmSIDER2a and LmSIDER2b: 1013 copies). Gaps (-) are introduced to maximize the alignments and conserved residues are shaded in grey.

**Fig. 5:**
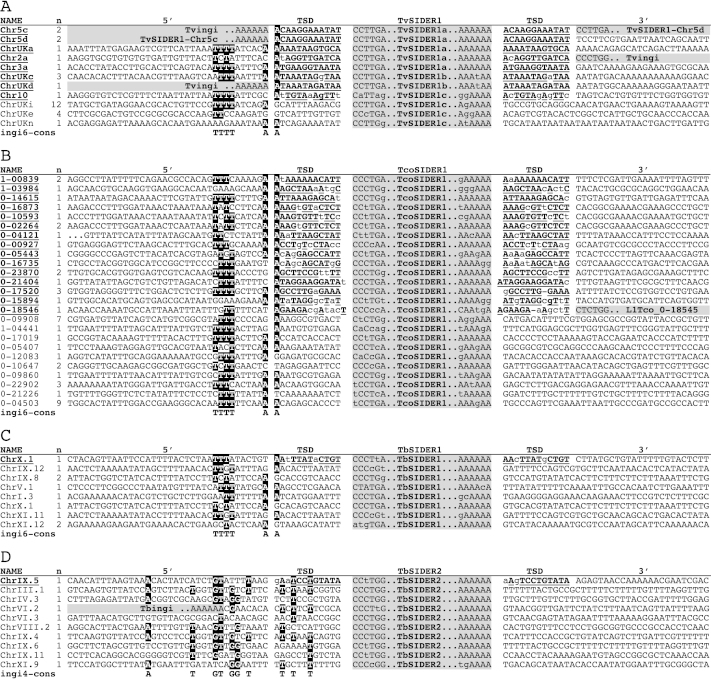
Comparison of the 5′- and 3′-adjacent sequences flanking the TSIDER1 (panels A–C) and TbSIDER2 (panel D) elements. Are considered only full-length SIDER elements flanked or not by a target site duplication (TSD) presenting 4 mismatches at the most. The name of the elements is indicated in the right margin. In panel B, numbers with six characters correspond to the contig number (0 or 1) followed by the position (without the 2 last numbers) of the retroposon in the *T. congolense* contig. The “n” column indicates the number of analyzed TSIDER elements flanked by nearly identical sequences. The alignment of all the selected sequences was based on the retroposon sequences (grey column headed “TvSIDER1”, “TcoSIDER1”, “TbSIDER1” or “TbSIDER2”) from which only the first and the last 6 bp, separated by the type of retroposons, are shown. The TSD flanking the retroposons is indicated by in bold-faced and underlined capital characters for the conserved residues; lower case characters in the TSD correspond to non-conserved residues. Residues within the TSD and 5′-flanking sequences that match the consensus observed upstream of the Tvingi and Tcoingi retroposons of the ingi6 subclade [12] (“ingi6-cons” in panels A–C) or the Tbingi retroposon of the ingi4 subclade [12,15] (“ingi4-cons” in panel D) are indicated in white characters on a black background. A grey background in the 5′- and 3′-adjacent sequences (“5′” and “3′”, respectively) means that the retroposon is preceded or followed, respectively, by another retroposon.

**Fig. 6:**
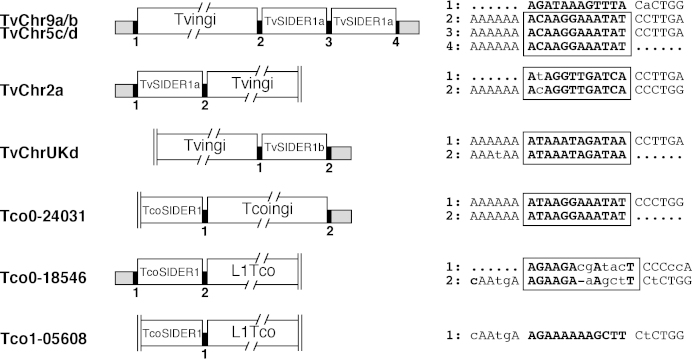
Comparison of the TSD flanking tandemly arranged retroposons in *T. vivax* and *T. congolense* genomes. The TvSIDER1, TcoSIDER1, Tvingi, Tcoingi and L1Tco retroposons (white boxes), the flanking regions (grey boxes) and the TSD (black boxes) are schematically represented. Vertical double bars indicate the end of the corresponding contig. TSD are indicated by numbered black boxes and the corresponding sequence is indicated on the right panel. Boxes define TSD, in which the nucleotides in bold face correspond to conserved residues. Only the first and the last 6 bp residues of the retroposons are indicated.

**Fig. 7:**
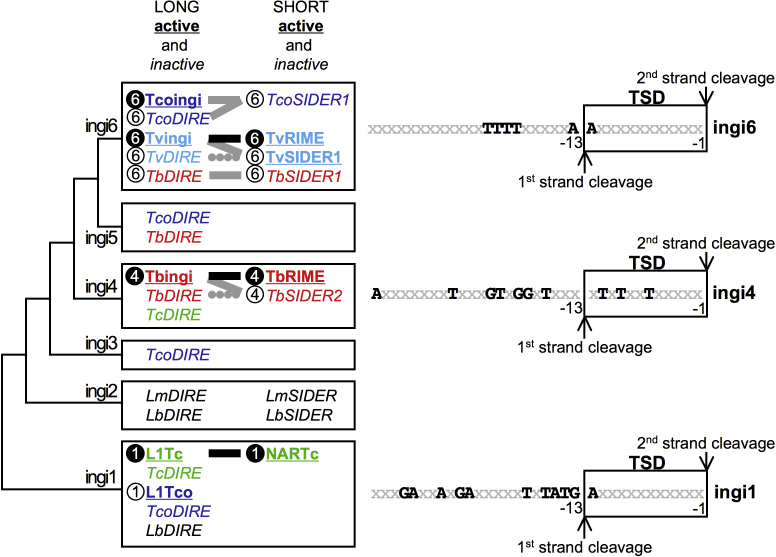
Relationship between long and short retroposons of the ingi clade. This figure shows a schematic representation of the phylogenetic tree constructed with the reverse transcriptase domain of protein-coding retroposons (long elements). The ingi subclade nomenclature (ingi1–6) was proposed before [12]. All known long or short retroposons of each subclade are shown in the central boxes (left and right side of the boxes, respectively), using a color code to distinguish between trypanosomatid species. Putative active elements in bold-faced and underlined characters showed evidence of recent mobilization, while inactive italicized elements are extinct ingi subclades not able to retrotranspose. Circled numbers represent the type of consensus sequence identified upstream of the retroposons, which is considered as the retroposon-encoded endonuclease binding domain. The original characterization of the consensus sequence (shown in the right part of the figure) is indicated by white numbers on a black background, such as the ingi1, ingi4 and ingi6 consensus upstream of L1Tc/NARTc [16], Tbingi/TbRIME [15] and Tcoingi/Tvingi/TvRIME [12], respectively. Thick black lines illustrate pairs of autonomous (long)/non-autonomous (short) retroposons, determined on the basis of sequence homology and 5′-conserved sequences. For thick grey lines, the proposed link between long and short elements is based only on 5′-conserved sequences. The dotted lines mean that the link between TvDIRE/TvSIDER1 and TbDIRE/TbSIDER2 is not clear because of limited data sets. The right panel shows the consensus sequence (bold-faced characters) upstream of ingi1, ingi4 and ingi6 retroposons. For the numbering, position −1 corresponds to the first residue upstream of the retroelement, the position of the first and second single-strand cleavages performed by retroposon-encoded endonucleases are indicated and the target site duplications (TSD) are boxed. (For interpretation of the references to color in this figure legend, the reader is referred to the web version of the article.)
