# Supplementary material for: TSIDER1, a short and non-autonomous Salivarian trypanosome-specific retroposon related to the ingi6 subclade
Source: Mol Biochem Parasitol. 2011 Sep;179(1):30–6. doi: 10.1016/j.molbiopara.2011.05.007 (PMC3820030; doi:10.1016/j.molbiopara.2011.05.007)
Supplement: Supplementary file 1 [file mmc1.doc]

Table S1. Retroposons of the ingi clade identified in the trypanosomatid genomes

| **Species** | **Name** | **Size (bp)** | **Gene**  **Product***^a^* | **Autonomous**  **- Active***^b^* | **Copy**  **Number***^c^* | **Ref** |
| --- | --- | --- | --- | --- | --- | --- |
| *T. brucei* | Tbingi | 5250 | 1657 | Auto - Active | 115 | [15] |
|  | TbDIRE | ~5000 | *nd^d^* | Auto - active | 73 | [21] |
|  | TbRIME | 500 | NC*^e^* | Auto - Active | 86 | [15] |
|  | **TbSIDER1** | **571** | **NC** | **Auto - Active** | **10** | [22] |
|  | TbSIDER2 | 573 | NC | Auto - Active | 12 | [22] |
| *T. congolense* | Tcoingi | 5404 | 1751 | Auto - Active | 56*^f^* | [12] |
|  | LITco | 4733 | 1505 | Auto - Active | 12*^f^* | [12] |
|  | TcoDIRE | ~5000 | *nd* | Auto - active | 173*^f^* | [12] |
|  | **TcoSIDER1** | **570** | **NC** | **Auto - Active** | **70*^f^*** |  |
| *T. vivax* | Tvingi | 5419 | 1752 | Auto - Active | 756*^g^* | [12] |
|  | TvDIRE | ~5000 | *nd* | Auto - active | 108*^g^* | [12] |
|  | TvRIME | 1030 | NC | Auto - Active | 58*^g^* | [12] |
|  | **TvSIDER1** | **576** | **NC** | **Auto - Active** | **32*^g^*** |  |
| *T. cruzi* | L1Tc | 4736 | 1524 | Auto - Active | 320 | [16] |
|  | TcDIRE | ~5000 | NC | Auto - active | 257 | [21] |
|  | NARTc | 260 | NC | Auto - Active | 133 | [14] |
| *L. major* | LmDIRE | ~5000 | *nd* | Auto - active | 52 | [21] |
|  | LmSIDER | 550 | NC | Auto - Active | 1858 | [22] |
| *L. braziliensis* | LbDIRE | ~5000 | *nd* | Auto - active | 65 | [6] |
|  | LbSIDER | 550 | NC | Auto - Active | 1986 | [23] |

*^a^* Number of amino acids contained in the multifunctional protein encoded by the consensus sequence of autonomous and active retroposons.

*^b^* Autonomous retroposons are potentially coding for a protein responsible for their retro-transposition. Retroposons are considered active when bioinformatics analyses suggest recent retrotransposition events for most of the elements composing the family.

*^c^* Copy number per haploid genome.

*^d^* Not determined due to of the high sequence heterogeneity.

*^e^* Non coding retroposons.

*^f^* The copy number of each retroposon in the 41.8 Mb dataset (the size of the haploid genome is not known).

*^g^* The copy number of each retroposon in the 47.7 Mb dataset (the size of the haploid genome is not known).
